# Description and consequences of sexual violence in Ituri province, Democratic Republic of Congo

**DOI:** 10.1186/1472-698X-11-5

**Published:** 2011-04-19

**Authors:** Françoise Duroch, Melissa McRae, Rebecca F Grais

**Affiliations:** 1 Médecins Sans Frontières (Operational Centre Geneva), 78 rue de Lausanne, CH-1211 Genève 21, Switzerland; 2 Epicentre, 53-55 rue Crozatier, 75012, Paris, France; 3 Harvard Humanitarian Initiative, Cambridge, Massachusetts, USA; 4 Geneva Center for Education and Research in Humanitarian Action (CERAH), University of Geneva, rue Rothschild, CH-1211 Genève 21, Switzerland

## Abstract

**Background:**

The war in eastern Democratic Republic of Congo has been the subject of numerous studies related to the problem of sexual violence. Historically, such violence is known to be part of strategic war plans to conquer and destroy communities, but it is now unfortunately prevalent in times of relative calm.

**Methods:**

We describe the characteristics and consequences of sexual violence in Ituri province of Democratic Republic of Congo through the retrospective analysis of 2,565 patients who received medical care in the Médecins Sans Frontières sexual violence clinic in the capital of Ituri province, Bunia, between September 2005 and December 2006. Using a standardised questionnaire, we report patients' demographics, number and status of aggressor(s), forced detention and violent threats among other variables for all patients presenting for medical consultation after a sexually violent event during this period.

**Results:**

Ninety-six percent of our cohort were female and 29.3% minors, 18-29 years was the most represented age group. Acts of sexual violence (n = 2,565) were reported to be mainly perpetrated by men with military affiliations (73%), although civilians were implicated in 21% of crimes. The attack was perpetrated by two or more persons in over 74% of cases and most commonly perpetrators were unknown armed males, (87.2%). Male victims accounted for 4% (n = 103) of our cohort. Forty-eight percent of our patients reported being attacked whilst performing daily domestic duties outside the home and 18% of victims being detained by their perpetrators, the majority of whom were held for less than 2 weeks (61.6%).

**Conclusions:**

The characteristics of sexually violent acts in Ituri province during this period cannot be simply explained as a 'weapon of war' as described in the literature, meaning the use of sexual violence within a military strategy where it is employed under the orders of a commander to harm a particular community. Whilst the majority of aggressions were by armed men there was an important proportion in which civilian perpetrators were implicated. This type of violence has become part of the general characteristics of violence in this war-torn population. Sometimes, as a means for some military factions to acquire remuneration with impunity and for some civilians, a means to counteract confronting, changing social norms occurring during chronic conflict.

## Background

For over a decade the population of the Democratic Republic of Congo (DRC) has been subject to an unrelenting humanitarian crisis; a local manifestation of a complex regional conflict that began in 1996 and continues to involve a collection of countries and multiple groups of armed combatants [1]. Spilling over the border from the 1994 Rwandan war and genocide, Hutu refugees and different rebel militia fractions arrived in eastern DRC, then Zaire. The latter engaged in ethnic clashes against the local Tutsi population and other ethnic fractions, and over the duration of the crisis, all parties have formed and reformed numerous armed alliances with local and foreign troops. Many accounts of this drawn out conflict demarcate two separate wars, the "First Congo War", 1994-1997, and the "Second Congo War" 1998-2002. Some estimates place the death toll from the "Second War" to be approximately 5.4 million [2].

The most recent war officially ended in December 2002 and a transitional government was put in place in June 2003 with ongoing peace negotiations the following years [3,4]. During these negotiations, several smaller conflicts have erupted across five eastern provinces resulting in clashes among armed groups and against civilians [5]. A series of rebel groups linked to foreign powers, predominately Uganda and Rwanda, competed to control this eastern area [5]. A 3-month retrospective mortality survey performed by Médecins Sans Frontières (MSF) in 2004-2005, in the eastern province of Ituri indicated a crude mortality rate (CMR) of 4.1 deaths/10,000/day (95% CI: 2.8-5.4) [6]. A DRC nationwide survey in 2006-2007 reported the national CMR to be 57% higher than the average rate for sub-Saharan Africa (2.2 deaths per 1,000 per month) [3].

Since the end of the "Second War", the fighting in eastern DRC has been increasingly associated with high rates of sexual violence that, at times, have been referred to as a 'plague' or of epidemic proportions [7]. Exact accounts are difficult to accurately estimate and individual reports quote differing yet staggering figures; a UN Population Fund study in 2006 found that 50,000 rape cases had been reported across nearly half of the health centres it the country [8]. This estimation is likely a mere fraction of the total.

One report concludes that in any given community up to 80% of women have been raped [9]. According to United Nations Population Fund data published in "Figures on sexual violence reported in the DRC in 2008", 15,996 new cases of sexual violence in DRC were reported that year; in 65% of cases adolescent girls were the targets of such crimes and 10% occurred in children under the age of 10 years [10]. Missing from these figures are the many thousands of episodes that remain unreported from victims who are unable, too scared or too ashamed, to seek assistance and be counted [10]. In one estimate, only 1 in 30 victims officially report sexual aggression and more than 50% of victims are unable to access healthcare either due to lack of healthcare services or unwillingness to seek care for fear of social stigmatisation [9].

For the majority of survivors, the medical and psychological consequences of sexual violence remain untreated thereby exposing victims to further suffering, potentially through sexually transmitted diseases, obstetric and gynaecological complications and untreated trauma injuries. Victims are additionally subjected to the psychological trauma resulting from being detained for varying lengths of time, subject to gang rape or sexual slavery and forced to witness sexual aggression towards their own family and community members. Congolese law, as well as social norms, have defined women as subordinate to males despite fulfilling the role of major support for the family [1]. The trauma of sexual violence is rarely treated as a 'war wound' and female victims suffer considerably in all aspects life from the related shame and stigma of the attack.

The concept of sexual violence being a 'weapon of war' and a 'social' action rather than 'individual' motivation is well described in the English scientific literature [9-13]. The term 'weapon of war' indicates the use of sexual violence within a military strategy where it is employed in order to harm a particular community under the orders of a commander [10]. A strategy used to terrorise populations and undermine social order with the aim of destabilising enemy communities [5,9,14]. Sexual violence has been employed as a tool of war on an unprecedented scale by virtually all forces involved in the DRC wars [14]. The simplicity of rationalising the epidemic of sexual violence in DRC to a single dimensional "weapon of war" strategy may be questioned now that the end of formal hostilities has not brought a reduction in the number of rape cases [15].

During the 2006 peace negotiations, the frequency of sexual aggression increased with the main perpetrators being reintegrated ex-combatants and regular civilians [10,15]. One explanation by Gingerich and Leaning (2004) focuses on the psychosocial and economic background theories behind the use of wartime sexual violence from case studies in DRC, Darfur, Rwanda, Kosovo and Chad [9,16]. This work highlights that non-military rape is more common in "highly communalized wars, where the division between civilian and combatant has collapsed and widespread hatred of an ethnic group have been allowed to prevail."

## Methods

Here, we describe the characteristics and consequences of sexual violence in Ituri province of DRC through the retrospective analysis of 2,565 patients who received medical care in the MSF sexual violence clinic in capital of Ituri, Bunia, between September 2005 and December 2006.

### Study site

**Figure 1:**
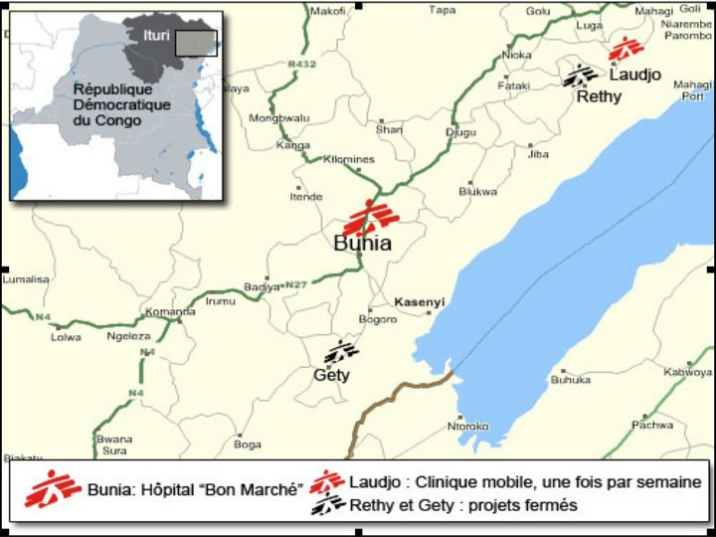
Map of Ituri province, Democratic Republic of Congo

Médecins Sans Frontières (MSF), Operational Centre Geneva, is an international non-governmental humanitarian organisation (NGO) whose staff has been working alongside the population of Ituri since 1999 and in Bunia since 2003. The province of Ituri lies in the north-eastern part of DRC, bordering Uganda and Sudan, with an estimated 4.6 million inhabitants [17]. Bunia is the province capital and also the largest city. In 2006, MSF clinics worldwide treated approximately 11,000 victims of sexual violence, 60% of whom sought care in Eastern DRC with Liberia and Burundi being the other countries with noticeably high numbers of patients seeking consultations (Figure 1).

In 2003, MSF set up "Bon Marché" hospital in Bunia during the peak of the humanitarian crisis. The program objectives were to provide primary healthcare (inpatient and outpatient) and emergency surgical care for the local populations who were the target of violent attacks. In July of that first year, specialised medical services for victims of sexual violence were implemented in response to the increasing need. Adjunct psychological services were integrated the following year in conjunction with other actors in the area. A rise in consultations relating to sexual violence occurred during renewed fighting in 2006 and in September of that year, MSF commenced treating victims of sexual violence in the main displacement camp in the region, Gety. Since 2009, the multiple medical services available at "Bon Marché" hospital are being transferred to the general referral hospital in Bunia. Other Congolese and international NGOs are present in Bunia with presence fluctuating depending on the security situation. During the time period of data collection MSF was the only NGO providing comprehensive medical care for victims of sexual violence in the area.

Our retrospective cohort study of 2,565 patients includes individuals who were victims of sexual violence between 1998 and 2006 and presented for care at "Bon Marché" Hospital or Gety Clinic between September 2005 and December 2006. Using convenience sampling, the data for analysis was collected during medical care consultations conducted within the MSF clinics and patients did not require an appointment in order to receive care free of charge. Medical consultations were conducted in a private environment and patient's recall of the incident recorded by Congolese medical personnel, predominately female, in general before the clinical examination. For the purposes of this report "sexual violence" predominately refers to rape but includes "any act of a sexual nature which is committed on a person under circumstances which are coercive", this includes forced vaginal and anal intercourse, oral sex, penetration with foreign objects and forced undressing in public [12]. Gang rape refers to sexual assault by two or more aggressors.

Quantitative data was extracted from interview records and included basic patient demographics, number and status of aggressor(s), and associated violent elements; threats, detention, possession of a weapon etc. Some 400 (15%) files were randomly selected for qualitative data analysis; theses files were selected according to file number, randomised via a computer-generated list. The collection of qualitative data was reviewed in order to find common threads amongst the patient histories and then categorised accordingly. STATA version 10 was used for quantitative statistical analysis.

We used existing program data from the MSF program, which was conducted in coordination with the Ministry of Health via a memorandum of understanding, which is the usual procedure for NGOs operating in these contexts. Data was collected during routine patient care visits, no supplementary interventions were conducted for the analysis presented. All electronic data were entered anonymously and identifiers were coded. No ethnic or identifying information was entered.

Our data has been classified to include a category referring to aggressors who belonged to an 'armed group'. This definition is favoured in DRC as the traditional use of the word 'soldier 'is difficult to define given the diversity of the origin of armed attackers including militia, military armed forces, peacekeeping troops and bandits. The term "known armed male" is used to refer to males, personally known to the victims who were armed during the aggression and includes armed civilians.

## Results

### Patient characteristics

The victims of sexual violence in this sample ranged from 1 year (n = 4) to over 50 years of age, mean age 25.7 years. Eighteen to 29 years was the most frequently represented age group (41%) and 29.3% of victims were minors referring to those less than 18 years of age. The majority of victims were female with males accounting for only 4% of victims (n = 103) and males were also most likely to be aged between 18 to 29 years at the time of presentation (Table 1).

**Table 1:** Demographics of patients presenting after sexually violent attack, according to age and sex

	Sex					
			
Age	Female		Male		Total	
	**N**	**%**	**n**	**%**	**n**	**%**
	
**Under 5 years**	29	1.1	1	0.01	30	1.2
**5 - 9 years**	54	2.1	0	0.0	54	2.1
**10 to 17 years**	604	23.6	3	0.1	607	23.7
**18 to 29 years**	1008	39.2	54	2.1	1062	41.3
**30 to 49 years**	661	25.8	30	1.2	691	27.0
**Over 50 years**	106	4.1	15	0.6	121	4.7

**Total**	**462**	**96.0**	**103**	**4.0**	**2565**	**100.00**

Approximately 80% of clinic presentations occurred within one year of the attack, mean time interval was 353.9 days. Only 24.5% sought care within the first week. Overall, for 73% of the victims, the reported attacker was an unknown armed male who they recalled being affiliated with a military or armed organisation. In the male victim subgroup, unknown armed males were reported in 95.2% of aggressions. A minority of male and female patients reported the aggressor to be a known, armed male (3.0%). In 21.6% of cases civilians were identified as perpetrators. The victims said they knew their perpetrator in only 13.7% of cases and in such cases; the violator was reported to be more likely to be an unarmed civilian who violated a minor (62%) (Table 2). In the incidences where the reported perpetrator was an armed male, 32.5% of victims could not remember the exact military affiliation. Of the 67.5% of victims who could recall this information, 62.5% of perpetrators were identified as being from one of three recognisable military armies and not an armed bandit group. A minority (2.5%) were reported as unable to recall of the identity of their perpetrator, all patients in this category were female.

**Table 2:** Identity of perpetrator according to victim age group and sex

Identity of Perpetrator												
**Age of victims**	**Known Civilian**		**Unknown civilian**		**Known Armed** **Male**		**Unknown Armed** **Men**		**Unable to recall**		**Total**	

	**n**	**%**	**n**	**%**	**n**	**%**	**n**	**%**	**n**	**%**	**n**	**%**
	
**Under 5 years**	26	1.0	1	0.0	0	0.0	0	0.00	3	0.1	30	1.2
**5 - 9 years**	42	1.6	5	0.20	3	0.1	3	0.12	1	0.0	54	2.1
**10 to 17 years**	120	4.7	75	2.9	29	0.3	370	14.4	13	0.5	607	23.7
**18 to 29 years**	61	2.4	107	4.2	35	1.4	837	32.6	22	0.9	1062	41.4
**30 to 49 years**	25	0.1	75	2.9	6	23.4	565	22.0	20	0.8	691	26.9
**Over 50 years**	4	0.15	14	0.55	3	0.1	97	3.8	3	0.1	121	4.7

**Total**	**278**	**10.8**	**277**	**10.8**	**76**	**3.0**	**1872**	**73.0**	**62**	**2.4**	2565	100.00

### Characteristics of sexual violence events

A total of 74.5% of victims (n = 1,912) were subjected to gang rape by multiple perpetrators at the same time, including 89.3% of male and 73.9% of female victims. Attack by between 2 to 4 perpetrators being the most common scenario (58.9%) for both sexes. Gang rape was reported in 55.7% (n = 385) of minors and thus more common that single perpetrator rape in all age groups. Of the victims who identified their perpetrator to be an unknown armed male, gang rape was reported by 87.2% compared with 54.5% of assaults by an unknown civilian and 16.9% by a known civilian. Gang rape involving more than 9 males was reported by 10 patients (0.3%) and only 1 patient was unable to recall information regarding the number of perpetrators.

Overall, 48.6% of victims (n = 2,565) were reportedly attacked whilst performing daily domestic activities outside the home, (i.e. the collection of water or firewood) and most often by unknown armed men although, this was the activity also associated with the highest frequency in civilians perpetrators (Table 3). The next most common locations were whilst victims were fleeing and/or displaced (18.2%), or in their own home (12.3%). Unknown armed men were implicated in 94.9% and 70.9% of cases respectively.

**Table 3:** Place of occurrence of sexual violence according to type of perpetrator for patients

Location of sexual aggression														
**Type of Perpetrator**	**Whilst performing outside domestic duties**		**Going or returning to school**		**Other locations**		**In the home**		**Whilst fleeing or displaced**		**Unable to recall**		**Total**	

	**n**	**%**	**n**	**%**	**n**	**%**	**n**	**%**	**n**	**%**	**n**	**%**	**n**	**%**
	
**Known civilian**	103	4.0	19	0.7	80	0.3	57	2.2	2	0.1	17	0.6	**278**	**10.8**
**Unknown civilian**	180	7.0	13	0.5	56	2.2	15	0.6	11	0.4	2	0.1	**277**	**10.8**
**Known armed male**	34	1.3	4	0.1	16	0.6	15	0.6	6	0.2	1	0.0	**76**	**2.9**
**Unknown armed male**	897	35.0	28	1.1	237	9.2	225	8.8	445	17.3	40	1.6	**1872**	**73**
**Details unknown**	35	1.4	2	0.1	11	0.4	5	0.2	5	0.2	4	0.2	**62**	**2.4**

**Total**	**1249**	**48.7**	**66**	**2.6**	**400**	**15.6**	**317**	**12.4**	**469**	**18.3**	**64**	**2.5**	**2565**	**100**

A total of 1,429 patients recalled having memorable threats made towards them during the act of violence. Mainly adult victims recalled such threats, 80.5% of whom were threatened with immediate death. Additional threats of further violence or death if the victim later spoke out were clearly remembered by 21.7% of patients, demands of money or goods (15.2%) or accusation of being affiliated with a military party or group (6.5%) were also vividly recalled. Of patients included in the sample for qualitative analysis, 45 patients (11.3%) remember the rapists saying they loved the victim or that the attack was an act of love and in at least 10 cases (2.5%) the women were raped as punishment for not having any money to give the attacker (Additional File 1).

A total of 2,290 patients recounted being subjected to a primary violent act and approximately 40% complained of more than one violent act occurring (Table 4). The most frequently reported associated violent act was being beaten (56.1%) followed by being violated in the presence of a close friend or relative (11.4%).

**Table 4:** Associated violence: First recalled response from 2152 patients

Associated Violence	n	%
Beaten	**1208**	**56.1**
Loss of property	**260**	**12.1**
In the presence of a close friend or relative	**245**	**11.4**
Witnessed a relative being subjected to physical violence	**14**	**0.6**
Forced to work	**80**	**3.7**
Forced to be a wife	**86**	**4.0**
Violated on several occasions	**54**	**2.5**
A man forced by the assailants to have sex with another female	**26**	**1.2**
Threat of Death	**44**	**1.0**
Report of the murder of a close friend or relative	**17**	**0.8**
Violated in public	**5**	**0.2**
Witnessed the murder of a close friend or relative	**17**	**0.8**
Sustained an injury from a weapon	**8**	**0.4**
Reported that physical violence was inflicted on a close friend or relative	**3**	**0.1**
A husband forced by the aggressor rape his wife	**8**	**0.4**
Introduction of foreign objects into the vagina or anus	**1**	**0.0**
Others	**75**	**3.5**

**Total**	**2152**	**100.0**

Detention or kidnapping following the attack was recalled by 18.5% of patients (n = 475), the majority were female with only 14.1% of detainees being male. A total of 32.6% of reported detainees were minors, of which only 2 (1.3%) were male. In 61.6% of cases they were held for less than 2 weeks and 3.8% were detained for longer than one year. Of the victims who recalled detention, 66.7% reported coming under attack whilst performing domestic duties away from their home or whilst fleeing for other reasons.

### Social Consequences

The social consequences of being a victim of sexual violence varied considerably among patients but included rejection by the family in 2.7% of cases in which the family was notified. Of interest, the rate of rejection between male and female victims did not differ greatly, 1.9% and 2.8% respectively, although 8.3% of females (compared to 1.9% of males) elected not to notify their family presumably from fear of rejection or stigmatisation. Another very relevant consequence of rape is pregnancy. This occurred in 88 of 245 (35.9%) females in whom this particular data was obtained.

## Discussion

Historically, discussions surrounding the phenomena of sexual violence in DRC have focused on the use of rape as part of a strategic warfare plan and an effective weapon for terrorising enemy communities and to destroy completely the social, family fabric of society [13]. Descriptive analysis of data collected from victims in Ituri province during 2005 and 2006 documents sexual violence, predominately rape, is being executed by a wider range of perpetrators. Our results confirm the well-recognised view that women are the significant victim group but we also note a small percentage of victimisation towards males who made up approximately 4% of our cohort. Whilst all figures are likely under-reported due to associated stigmatism, male rape is more difficult to investigate and accurately report, as they have traditionally been more reluctant to report events of sexual violence [18].

In our data, armed men were reported to have committed the majority of sexual crimes (73%) although in approximately 32% of these cases, victims could not recall their affiliated military fraction. For victims who did recall the affiliated fraction of their aggressor, almost two-thirds came from one of three recognisable armies. In our cohort, the largest group of women reported being attacked whilst performing daily domestic activities outside the home. This trend correlates with other patterns seen in contexts with large-scale sexual violence during conflict such as Darfur where 82% of rapes occurred whilst women were outside populated villages/towns whilst searching for firewood [19]. A recent retrospective study also conducted in eastern DRC reported contrasting results with 57% of women attacked in their homes, often while sleeping with families [14].

Our analysis supports the widely held view that gang rape persists and is a frequent mode of rape in DRC. Over 70% of victims in our sample reported being subject to sexual assault by multiple perpetrators, most frequently involving 2-4 perpetrators. In the large majority of reported gang rape cases, victims identified the aggressor to be an unknown armed male. The latter may suggest a male linked with a military fraction. These results are in keeping with other published literature on rape in this part of the world; one retrospective study of 492 female rape survivors indicated that 71% were gang raped whilst a sample of 1,851 rape survivors in DRC reported a lower frequency of gang rape (60%) in patients presenting to hospital after sexual violence although the mean number of perpetrators was comparable with our results (mean = 2.1) [2,14].

Interviews conducted with military personnel who have performed such acts, estimate that a number of the aggressions are committed in relation to individual motivations as well as frustrations related to the environment of war, poverty and conflict [16,20]. A paper by Baaz and Stern (2009) discussed soldier's perspectives on why rape occurs during wartime in DRC [20]. The soldiers' justifications were broadly classified into 2 major categories; 'Lust Rape' carried out by deprived soldiers who lust for sex but have no money and no leave to see their families, and 'Evil Rape', which is connected to brutality, mutilation and killing and motivated by a desire to humiliate peoples' dignity and not sexual desire. Some soldiers referred to the former as 'normal' rape, apparently justified within the "craziness" of wars that disrupt society organisation, and represented as possibly morally 'acceptable' as a man must release sexual tension. The soldiers reported that 'Evil rape' is different to 'Lust Rape' or 'normal' rape. It is linked to a sense of moral disengagement and is not considered acceptable by soldiers although, at times 'understandable' as soldiers are 'ordered' to rape and feel a "general wish to destroy" which stems from "suffering and "frustrations" [15]. In the case of 'normal' rape, the aggressor can justify himself being exonerated from the crime, as he himself is a victim of circumstance [20].

In contrast, Congolese women have been asked their opinion of the motivation behind such sexual crimes and 83% indicated that lack of organisation, training and discipline among the armed parties was a contributing factor [2]. Other internal MSF data reports that 57% of victims felt rape was used as a "deliberate method of extermination the Congolese people" [2]. There are also increasing numbers of men and boys reporting sexual assault by combatants [5]. Male victims accounted for 4% of our cohort and armed men were identified as perpetrators in 95.2% of those male sexual assault cases.

Importantly, analysis of this dataset demonstrates the significant contribution of civilians in continuing to perpetrate these sexual crimes. This is recognised as an increasing phenomenon in the context of DRC in recent literature [10,18]. Project data from Burundi has documented an increase in monthly rape cases after withdrawal of armed groups from conflict areas; perpetrators included neighbours, relatives and local burglars [15]. We have documented that over 1 in 5 of the victims of sexual violence presenting to MSF clinics reported being violated by civilians; including civilians either no longer, or potentially never, affiliated with an 'armed group'. Additionally, another 3% of patients reported being assaulted by a known armed male who may be self-armed rather than associated with a military fraction and thus could potentially further increase the contribution of civilians. Other published retrospective work reports less than 1% of sexual crimes were perpetrated by civilians in 2004, increasing to 38% in 2008. Of note, our dataset reports a much higher frequency relative to the timeframe 2005-2006 [18].

In focus group reports, some Congolese men have "acknowledged that rape has become the norm for young men growing up during conflict in Eastern DRC" [18]. This changing conduct may be secondary to changes that have occurred in communities enduring chronic conflict with 20 years of war in DRC having caused a shift in social norms [18]. Firstly, family life has changed; males, and many children, have involuntarily served as combatants leaving women responsible for driving family economics and vulnerable to attack and kidnapping whilst at home alone. Left in the position of economic drivers, some women have resorted to selling sexual favours to provide for their household [21]. Such actions increase the distance between men and women and helps explain the position of each gender in the current crisis and the motivations behind rape [21]. Secondly, males are no longer able to fulfil their traditional role as provider for the family and seek to exert their power in other ways [20]. Finally, ordinary civilians have armed themselves against attack creating an ease of access to weapons that fuels violence within the community and sexual violence has proliferated to the point of being used in minor transgressions or to settle old personal scores and tribal disputes [13,15].

This is a multi-dimensional issue. Society has digressed from previously practised cultural traditions that punished offenders [13,15]. Criminal impunity for soldiers initially acting under the command of leaders may extend to all perpetrators of sexually violent crimes and tribal or local laws no longer hold the same value in community life. Furthermore, repatriation of foreign troops and increased movement of local militia enables local, civilian perpetrators to continue to use the presence of foreigners' as cover to attack with impunity [13]. This creates the opportunity for communities to be left without effective means to reduce the violence or hold the perpetrators to justice leading to further disintegration of the moral and social fabric [22].

The study had several limitations, firstly the analysis is retrospective from patient data files and original patient information cannot be clarified or verified. The limitations associated with patient recall and known under-reporting of sexual violence cases in such contexts are also identified, particularly in the male victim subgroup. Male medical staff were not always available for consults at the MSF clinic and this may have deterred male victims from seeking care. We acknowledge the limitations of establishing a real epidemiology of sexual violence in DRC only through 'presentation to clinics', and have seen that there is limited association between the timing of the attack and presentation at health facility, as often women present later during times of improved security.

## Conclusions

Acts of sexual violence, predominately rape, continue in DRC with civilians as the reported perpetrators of more than 1 in 5 of the aggressions in our cohort. The incidence of sexual violence is not diminishing with the withdrawal of armed forces. Changing social norms secondary to chronic war appear to have influenced community life and had an impact on the traditional male to female relationship in DRC [18]. In the region we studied, social change may be linked with the reported increase in civilians perpetrating these crimes, compared to earlier accounts of rape being used primarily as a "weapon of war". The significant proportion of victims continues to be women, aged 18 - 29 years who report being attacked whilst performing domestic duties away from the home, only a minority recall attack within the home. The social repercussions for female victims persist with approximately one third of females questioned becoming pregnant as a result of rape, and 8.3% not notifying family of the crime compared to 1.9% of male victims.

The true extent of these crimes will never be known, as patients presenting for treatment fear the stigmatisation, loss and potential excommunication of their community and/or family. Despite the acknowledged limitations of this descriptive analysis, these results should help to encourage the continuing research on gender issues in the region.

## Competing interests

The authors declare that they have no competing interests.

## Authors' contributions

FD arranged data collection and takes responsibility for the accuracy of the data. MM completed data analysis and in conjunction with RG participated in interpretation of results. MM, RG and FD participated in the writing and RG and MM performed critical revision of the manuscript. All authors have read and approved the final manuscript.

## Pre-publication history

The pre-publication history for this paper can be accessed here:

http://www.biomedcentral.com/1472-698X/11/5/prepub

## Supplementary Material

Additional file 1 **Phrases remembered by victims if they refused rape**. A selection of free text phrases that the victims recall the perpetrator(s) saying to them during the act of aggression. Click here for file
