# Supplementary material for: Description and consequences of sexual violence in Ituri province, Democratic Republic of Congo
Source: BMC Int Health Hum Rights. 2011 Apr 19;11:5. doi: 10.1186/1472-698X-11-5 (PMC3108309; doi:10.1186/1472-698X-11-5)
Supplement: Additional file 1 — Phrases remembered by victims if they refused rape. A selection of free text phrases that the victims recall the perpetrator(s) saying to them during the act of aggression. [file 1472-698X-11-5-S1.DOC]

**Additional files**

### Phrases remembered by victims if they refused rape

| Sample threats of violence or death if the victim resisted rape |
| --- |
| n° 110 ‘Choose between life and death’ |
| n° 123 ' If you refuse we will kill you’ |
| n° 130 'If you refuse you will be dead’ |
| n° 136 ‘We need your body for sex, if you refuse you are going to find yourself dead’ |
| n° 138 'If you refuse to sleep with us you will be dead’ |
| n° 140 'Undress yourself immediately if you do not want to die’ |
| n° 157 'Between life and death, what is your choice’ |
| n° 159 'Today you are at my disposal whether you like it or not, otherwise I will terminate you’ |
| n° 167 'We need your body, otherwise we will kills you’ |
| n° 175 ‘Now you are going to become my wife and if you dare refuse I will kill you immediately’ |
| n° 197 ‘Sleep with us otherwise we kill you all’ |
| n° 198 ‘Whether you want it or not we are going to sleep with you otherwise we kill you’ |
| n° 202 ‘Lay down on the ground for me to have sex with you or I will kill you’ |
| n° 206 ‘We want to sleep with you otherwise we will eliminate you’ |
